# The Effects of Different Exercise Modalities in the Treatment of Cardiometabolic Risk Factors in Obese Adolescents with Sedentary Behavior—A Systematic Review and Meta-Analysis of Randomized Controlled Trials

**DOI:** 10.3390/children8111062

**Published:** 2021-11-18

**Authors:** Daxin Li, Ping Chen

**Affiliations:** Department of Physical Education, Ocean University of China-Laoshan Campus, Qingdao 266100, China; lidaxin@stu.ouc.edu.cn

**Keywords:** exercise, cardiometabolic risk factors, obese adolescents, sedentary behavior, meta-analysis

## Abstract

Purpose: Obesity has become increasingly prevalent in adolescents due to unhealthy diet habits, sedentary behavior and a lack of physical activities. This study aims to assess the effects of different exercise modalities in the treatment of cardiometabolic risk factors (CRF) in obese adolescents with sedentary behavior. Methods: A systematic search was conducted using databases (PubMed, Embase, Cochrane library, Web of Science, CNKI and VIP database) from the earliest available date to August 2021. Nineteen randomized controlled trials (RCTs) with 704 participants were included. The included studies were evaluated for methodological quality by the Cochrane bias risk assessment tool, and a statistical analysis was performed by the Review Manage 5.3 and Stata 15.1 software. Results: The results of the meta-analysis showed that exercise could significantly improve obese adolescents’ body mass index (BMI) (MD = −1.99, 95% CI: −2.81 to −1.17, *p* < 0.00001), low density liptein cholesterol (LDL-C) (SMD = −0.98, 95% CI: −1.58 to −0.37, *p* = 0.002), triglyceride (TG) (SMD = −0.93, 95% CI: −1.72 to −0.14, *p* = 0.02), total cholesterol (TC) (SMD = −1.00, 95% CI: −1.73 to −0.26, *p* = 0.008), peak oxygen uptake (VO_2_peak) (MD = 3.27, 95% CI: 1.52 to 5.02, *p* = 0.0003) and homeostatic model assessment insulin resistance (HOMA-IR) (SMD = −2.07, 95% CI: −3.3 to −0.84, *p* = 0.001). However, there was no statistically significant difference in high-density liptein cholesterol (HDL-C) (SMD = 0.40, 95% CI: −0.28 to 1.08, *p* = 0.25). Conclusion: Exercise can effectively improve cardiometabolic risk factors in obese adolescents with sedentary behavior. For obese adolescents who want to lose weight and improve cardiorespiratory fitness, combined aerobic and resistance training and high-intensity interval training are optimal choices. For obese adolescents with high blood lipids, aerobic training can be regarded as a primary exercise modality to reduce the high risk of cardiovascular diseases; For obese adolescents with insulin resistance, combined aerobic and resistance training can be considered to reduce the high risk of diabetes. It is hoped that more high-quality studies will further expand the meta-analysis results and demonstrate the optimal exercise frequency and treatment intensity of cardiometabolic risk factors in obese adolescents with sedentary behavior in the future.

## 1. Introduction

Childhood obesity is one of the main challenges that public healthcare currently faces worldwide. The prevalence of obesity has doubled in the past decade, with an increasing trend in younger generations [1]. Obesity is associated with cardiovascular and metabolic risk factors, such as diabetes mellitus, coronary artery disease, dyslipidemia and hypertension, which is a major public health concern in adolescents [2]. In addition, obesity can also cause a series of psychological problems, such as anxiety and depression. A series of problems in obese adolescents can have a negative impact on life-long health.

Childhood obesity is mainly caused by sedentary behavior, insufficient physical activity and unhealthy diet habits [3]. Physical activity was suggested as a beneficial treatment to help prevent obesity as well as improve CRF in obese adolescents [4]. Several previous studies explored the effect of exercise intervention on obese adolescents [5,6,7,8]. Kim et al. [5] assessed the effect of a 12-week jump rope exercise program on body composition, insulin sensitivity and academic self-efficacy in obese adolescent girls, the conclusion showed that jump rope exercise intervention can be a useful therapeutic treatment to improve cardiovascular disease risk factors in obese adolescent girls. Bharath et al. [6] explored the effectiveness of combined resistance and aerobic exercise training on insulin resistance and central adiposity in obese adolescents; the results showed that the combined resistance and aerobic exercise was a useful therapeutic method to alleviate metabolic risk factors in obese adolescents. Son et al. [7] explored the effect of 12-week combined exercise training on blood pressure, arterial stiffness and insulin resistance in obese adolescent girls, the results showed that 12 weeks of combined exercise training improved blood pressure and insulin resistance and reduced the central adiposity in obese adolescent girls with prehypertension. Yin et al. [8] also found that physical activity with a frequency of at least 3 days/week was effective in reducing adiposity and improving CRF for obese adolescents. Lee et al. [9] held that combining resistance exercise with weight management interventions was a useful strategy for improving muscular fitness and reducing obesity-related risks in children and adolescents. Although some existing RCTs have explored the effectiveness of physical activity on CRF in obese adolescents, however, it’s unclear that the effect of different exercise modalities in the treatment of CRF in obese adolescents due to the different exercise mode and duration. Thus, this study is the first meta-analysis to explore the effect of different exercise modalities in the treatment of CRF in obese adolescents with sedentary behavior, the specific objectives were: (1) To explore the effect of different exercise modalities in the treatment of CRF in obese adolescents with sedentary behavior (sub-analysis with different exercise modalities and intervention duration. (2) To explore the optimal exercise modality for obese adolescents with different abnormal physiological parameters and provide reasonable exercise prescription.

## 2. Methods

This meta-analysis was performed according to the PRISMA (Preferred Items for Reporting of Systematic Reviews and Meta-Analyses) guidelines [10].

### 2.1. Search Strategy

Two investigators searched Pubmed, Embase, Cochrane library, Web of Science, CNKI and VIP Database. The Randomized Controlled trials (RCTs) were searched from the earliest available date to August 2021 using the following terms: (Pediatric Obesity OR Adolescent Overweight OR Adolescent Obesity OR Childhood Obesity OR Childhood Overweight) AND (Exercise OR Physical Activity OR Physical Training OR Physical Exercise OR Sport) without any limitations. Meanwhile, the references of studies included in relevant reviews were screened to identify other possible relevant studies. A detailed summary of the literature search is depicted in Table 1.

### 2.2. Study Selection

The inclusion criteria for this meta-analysis were full-text research (randomized controlled trials) articles, published in peer-reviewed academic journals, and written in Chinese or English. The exclusion criteria were: (1) Obese adolescents with cardiac diseases; (2) the outcome does not meet the requirements; (3) there is significant difference in the baseline values (*p* < 0.05).

All relevant studies were independently screened through reading titles and abstracts by two researchers. Irrelevant studies were excluded in the process of initial screening. Then, those studies that met the standards were collected and downloaded. Irrelevant articles were excluded by reading the full text. The disputes in the process of study selection were settled by discussion.

### 2.3. Quality Assessment

The quality of the included studies was assessed by two authors using Cochrane Handbook for Systematic Reviews of Interventions 5.0.1. Disagreements were resolved by discussion.

### 2.4. Data Extraction

Two researchers independently screened the studies by reading the titles and abstracts and excluded irrelevant studies. Finally, the articles that met the standards were included and synthesized. Differences in the assessment of study eligibility were resolved by discussion.

### 2.5. Statistical Analysis

Statistical analyses were performed using Review Manager 5.3 (Nordic Cochrane Centre, Copenhagen, Denmark) and Stata MP 15.0 (StataCorp, Pyrmont, Australia). For analysis of continuous variables, the mean difference (MD) or standardized mean difference (SMD) with 95% confidence intervals (95% CI) was used to pool effect sizes. Heterogeneity among studies was examined with Cochran’s Q and I^2^ statistic, in which values greater than 50% indicated significant heterogeneity and random-effects model was chosen [11]. The overall effects were considered significant when *p* ≤ 0.05. Subgroup analysis and sensitivity analysis were performed to explore possible effects on heterogeneity. Finally, Egger’s regression was performed to assess publication bias.

## 3. Results

### 3.1. Study Selection

**Figure 1:**
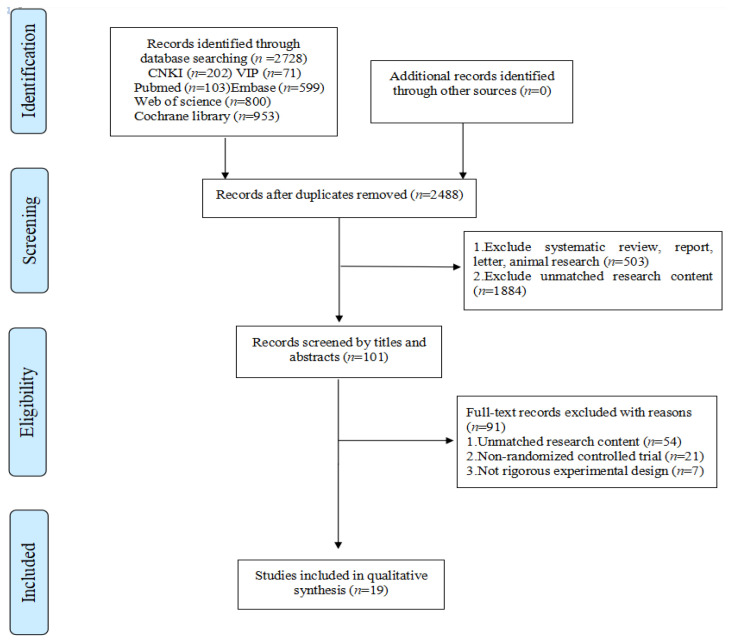
Flow Diagram of Study Selection.

The study identified a total of 2728 articles. One hundred and one citations were screened with full-text reading by two independent researchers, after duplicates, and irrelevant studies were removed. A total of 19 RCTs [5,6,7,12,13,14,15,16,17,18,19,20,21,22,23,24] were included in the meta-analysis. The selection process can be found in the PRISMA diagram in Figure 1.

### 3.2. Study Characteristics

The characteristics of the included studies are shown in Table 2. The included nineteen studies [5,6,7,12,13,14,15,16,17,18,19,20,21,22,23,24] involved a total of 704 obese adolescents. The age of the participants varied from 8 to 17 years. Intervention duration ranged from 8 to 20 weeks with a frequency of exercise training ranging from 2 to 7 days per week. Exercise time ranged from 23 min to 60 min. The participants in the experimental group all received exercise intervention, and participants in control group received non-intervention or education intervention.

### 3.3. Risk of Bias Assessment

**Figure 2:**
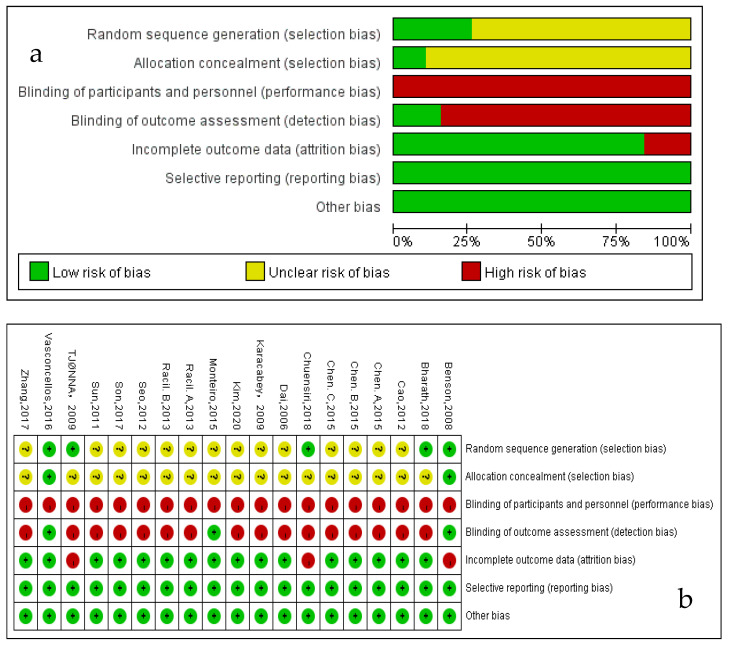
Risk of bias (**a**) Risk of bias graph: review authors’ judgements about each risk of bias item presented as percentages across all included studies; (**b**) Risk of bias summary: review authors’ judgements about each risk of bias item for each included study.

The risk of bias for the included studies was assessed with the Cochrane Risk of Bias Tool and the results are showed in Figure 2. Five studies [6,13,15,22,24] described the random sequence generation (selection bias), which was evaluated as low-risk. Two studies [13,22] had a low risk of bias in allocation concealment (selection bias) using a sealed envelope. Three studies [13,21,22] were blinded to the assessor (detection bias) and three studies [13,15,24] had incomplete outcome data (attrition bias). There was no reporting bias and other bias in the included studies.

### 3.4. Meta-Analysis

#### 3.4.1. Body Mass Index (BMI)

**Figure 3:**
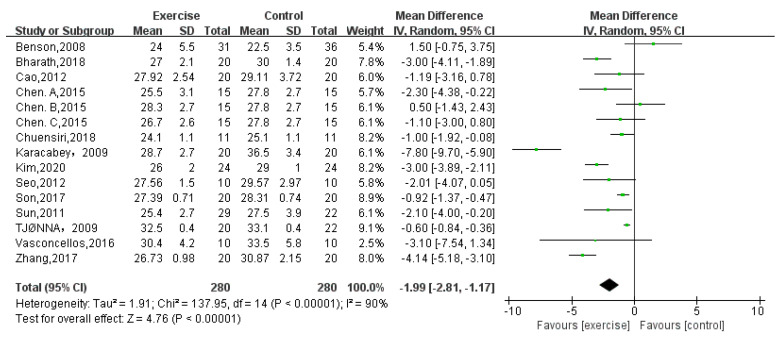
Forest plot of BMI.

Fifteen trials [5,6,7,13,14,15,16,17,18,20,22,23,24] with a total of 560 participants provided data on BMI. The meta-analysis showed a significant improvement for participants in the exercise group (random-effects model: MD = −1.99; 95% CI: −2.81 to −1.17; *p* < 0.00001) (Figure 3). The test for heterogeneity was significant (*p* < 0.00001; I^2^ = 90%).

#### 3.4.2. High-Density Liptein Cholesterol (HDL-C)

**Figure 4:**
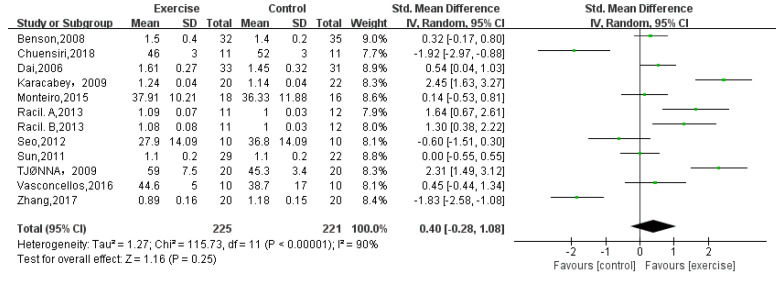
Forest plot of HDL-C.

HDL-C was reported by twelve studies [12,13,14,15,16,18,19,21,22,23,24] that included a total of 446 obese adolescents. The result showed no significance for obese adolescents (random-effects model: SMD = 0.4; 95% CI: −0.28 to 1.08; *p* = 0.25) (Figure 4). The test for heterogeneity was significant (*p* < 0.00001; I^2^ = 90%).

#### 3.4.3. Low-Density Liptein Cholesterol (LDL-C)

**Figure 5:**
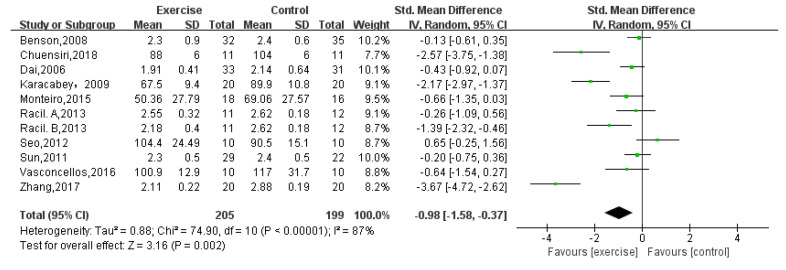
Forest plot of LDL-C.

LDL-C was reported by eleven studies [12,13,14,16,18,19,21,22,23,24] that included a total of 404 obese adolescents. The result showed a significant difference between exercise group and control group (random-effects model: SMD = −0.98; 95% CI: −1.58 to −0.37; *p* = 0.002) (Figure 5). The test for heterogeneity was significant (*p* < 0.00001; I^2^ = 87%).

#### 3.4.4. Triglyceride (TG)

**Figure 6:**
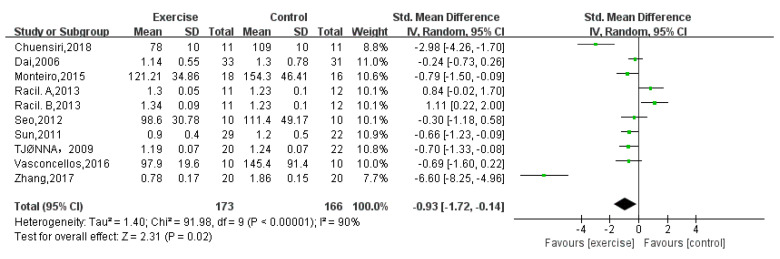
Forest plot of TG.

Ten studies [12,15,16,18,19,21,22,23,24] with a total of 339 obese adolescents reported a significant difference in TG (random-effects model: SMD = −0.93; 95% CI: −1.72 to −0.14; *p* = 0.002) (Figure 6). The test for heterogeneity was significant (*p* < 0.00001; I^2^ = 93%).

#### 3.4.5. Total Cholesterol (TC)

**Figure 7:**
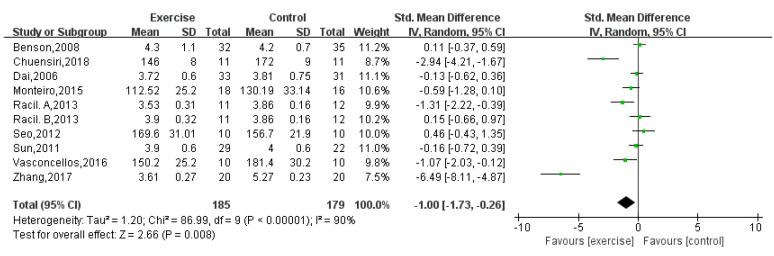
Forest plot of TC.

Ten [12,13,16,18,19,21,22,23,24] studies with a total of 364 obese adolescents reported a significant difference in TC (random-effects model: SMD = −1.00; 95% CI: −1.73 to −0.26; *p* = 0.008) (Figure 7). The test for heterogeneity was significant (*p* < 0.00001; I^2^ = 90%).

#### 3.4.6. Peak Oxygen Uptake (VO_2_peak)

**Figure 8:**
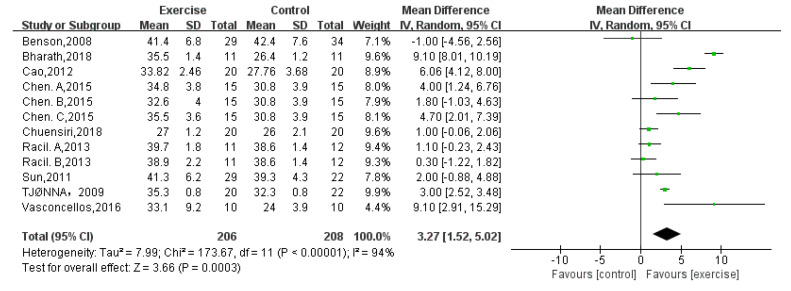
Forest plot of VO_2_peak.

VO_2_peak was reported by twelve studies [6,13,15,16,17,19,20,22,24] that included a total of 414 obese adolescents. The meta-analysis showed a significant difference between the exercise group and control group (random-effects model: MD = 3.27; 95% CI: 1.52 to 5.02; *p* = 0.0003) (Figure 8). The test for heterogeneity was significant (*p* = 0.0001; I^2^ = 80%).

#### 3.4.7. Homeostatic Model Assessment-Insulin Resistance (HOMA-IR)

**Figure 9:**
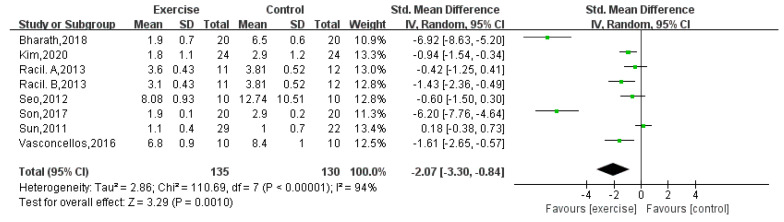
Forest plot of HOMA-IR.

HOMA-IR was reported by eight studies [5,6,7,16,18,19,22], including 265 obese adolescents. The aggregate results of these studies showed that there was a statistically significant difference between the two groups (random-effects model: MD = −2.07; 95% CI: −3.30 to −0.84; *p* = 0.001) (Figure 9). The test for heterogeneity was significant (*p* < 0.00001; I^2^ = 94%).

#### 3.4.8. Subgroup Analyses

Subgroup analyses, based on exercise modality and intervention duration, were performed to explore heterogeneity and the separate effects of different exercise modalities and intervention durations (Table 3). The results of the subgroup analyses showed that exercise modality and intervention duration were not the potential factors that led to heterogeneity for the outcomes of BMI, HDL-C, LDL-C, TG, TC, VO_2_peak, and HOMA-IR.

Meanwhile, the results of subgroup analyses showed that:

1. An intervention duration of ≥12 weeks had a greater advantage in improving obese adolescents’ BMI, HDL-C, LDL-C, TC, and HOMA-IR than an intervention duration <12 weeks.
2. Combined aerobic and resistance training (CART) and high-intensity interval training (HIIT) had more significant effects in reducing BMI and improving the VO_2_peak than aerobic training (AT) and resistance training (RT).
3. AT showed more significance in improving obese adolescents’ serum lipids than RT, CART and HIIT.
4. CART showed more significance in improving obese adolescents’ insulin resistance than AE and HIIT.

#### 3.4.9. Sensitivity Analysis

Sensitivity analysis was performed by removing each individual study one-by-one; the exclusion of individual studies did not substantially alter heterogeneity and the overall results.

#### 3.4.10. Publication Bias

Egger’s test was performed for the six outcomes (Table 4). There were no significant publication biases for BMI, HDL-C, LDL-C, TC, and VO_2_peak. However, there was a small publication bias for TG (asymmetry test, *p* = 0.01). In the future, more studies must be included in the analyses.

## 4. Discussion

The childhood obesity epidemic has become a serious and major public health problem worldwide. Obese children and adolescents are likely to remain obese during adulthood and are at higher risks of developing chronic diseases such as hypertension, type 2 diabetes, cancers, and cardiovascular diseases [25]. Previous studies assessed the effect of exercise on physiological indicators in obese adolescents [5,6,7,8]. However, there are no detailed exercise suggestions for different obese adolescents, which may have complex cardiometabolic risks. This meta-analysis included all the relevant studies [5,6,7,12,13,14,15,16,17,18,19,20,21,22,23,24], which contained different exercise modes and enough sample sizes and aimed to explore the optimal exercise modality for obese adolescents with different, abnormal, physiological parameters and provide a reasonable exercise prescription.

BMI is calculated by dividing an individual’s weight in kilograms by height in meters squared > 25 kg/m^2^. It is a major risk factor for a wide range of chronic diseases, including cardiovascular disease, type II diabetes, and some cancers [26]. The results of the meta-analysis showed that exercise could significantly reduce BMI in obese adolescents (MD = −1.99, 95% CI: −2.81 to −1.17, *p* < 0.00001). Meanwhile, similarly, the results of the subgroup analyses showed that CART and HIIT significantly improved obese adolescents’ BMI. The mechanism of exercise that improves BMI may be explained by three aspects: (1) Exercise can stimulate responses in the adipose tissue and promote free fatty acids in the blood to enter the cells, so as to improve the utilization ratio of adipose. (2) Exercise improves the activities of lipoprotein lipase and hepatic lipase in the muscle and liver, so as to accelerate lipocatabolic [27]. (3) Exercise can decrease leptin, tumor necrosis factor-α, and interleukin-6. Meanwhile, exercise can improve the expression of the uncoupling protein-3 mRNA in the skeletal muscle and the catecholamine level, which can increase the resting metabolic rate of obese adolescents and increase their energy consumption in the resting state, so as to promote the metabolism level of adipose in this state [28,29].

Blood lipids, which include HDL-C, LDL-C, TG, and TC, received attention in cardiovascular diseases [30]. HDL-C is a non-covalent, quasi-spherical complex of lipids that is inversely associated with the risk of cardiovascular disease [31]. The results showed that exercise could not effectively improve HDL-C for obese adolescents (SMD = 0.4; 95% CI: −0.28 to 1.08; *p* = 0.25); this conclusion is similar to previous meta-analyses that explored the effects of exercise on HDL-C in different populations [32,33]. Conversely, the results of the meta-analysis showed that exercise could significantly improve LDL-C (SMD = −0.98, 95% CI: −1.58 to −0.37, *p* = 0.002), TG (SMD = −0.93, 95% CI: −1.72 to −0.14, *p* = 0.02) and TC (SMD = −1.00, 95% CI: −1.73 to −0.26, *p* = 0.008) for obese adolescents. Moreover, AT showed greater advantages in improving blood lipids than RT, CART, and HIIT in obese adolescents. The mechanism of exercise that improves blood lipids mainly focuses on two aspects: (1) Exercise can increase the whole-body fat oxidation, adipose tissue lipolysis, and fatty acid utilization by skeletal muscle. It can also dictate intracellular lipid delivery and mitochondrial β-oxidation, so as to promote the muscle to absorb and use more free fatty acids and cholesterol [34,35]. (2) Exercise may increase the reverse transport capacity of TC, which may result in a decrease in LDL-C and an increase in HDL-C in blood. Some previous studies explored the potential physiological mechanism of exercise for improving participants’ blood lipids, but there is still no clear explanation. Thus, the mechanism of exercise for improving blood lipids requires further exploration.

The VO_2_peak is commonly used to evaluate cardiorespiratory fitness because the ability of the cardiopulmonary system to deliver oxygen to the exercising muscles is a critical limiting factor in determining this measurement, especially in sedentary populations [36]. The results of meta-analysis showed exercise can significantly improve VO_2_peak (MD = 3.27, 95% CI: 1.52 to 5.02, *p* = 0.0003). Meanwhile, the results of subgroup analysis showed that AT, CART and HIIT elicited similarly significant difference in improving VO_2_peak. Many previous meta-analyses assessed the effect of exercise on VO_2_peak [37,38]. Cao et al. [37] explored the effect of HIIT and AT on VO_2_peak in healthy children and adolescents, the conclusion reported that HIIT had greater improvements on cardiorespiratory fitness among healthy children and adolescents than AE. Lee et al. explored the effects of CART on VO_2_peak in patients with stroke, the results suggested that an exercise program consisting of moderate-intensity exercise, 3 days per week, for 20 weeks should be considered for greater effect on cardiorespiratory fitness in stroke patients [38]. The physiological mechanism of exercise that improves VO_2_peak mainly focuses on the following aspects: (1) Exercise changes the oxygen transport system and the ability of muscle to use oxygen, which is mainly manifested in the increase in capillary density around muscle cells, the improvement of muscle cell oxidation ability, and the enhancement of the activity of oxidase, such as citrate synthase, so as to enhance the ability of muscle to use phosphate, glycogen, and triglyceride [39]. (2) Exercise can slow down the resting heart rate and increase the stroke output of obese adolescents, as well as improve the heart pumping function and work efficiency, increasing the capacity of oxygen uptake and utilization [40].

Insulin resistance (IR) is the primary cause of type 2 diabetes and it occurs many years before the onset of type 2 diabetes in humans. IR occurs when the insulin-sensitive tissue loss responds to insulin [41]. The homeostasis model assessment of insulin resistance (HOMA-IR) is a proxy estimate of IR based upon the relationship between fasting glucose and insulin levels, with higher values of HOMA-IR representing more severe IR [42]. The meta-analysis results suggested that exercise showed a statistical significance in improving HOMA-IR (SMD = −2.07, 95% CI: −3.3 to −0.84, *p* = 0.001). The subgroup analysis results showed that CART was the optimal exercise mode for obese adolescents to improve HOMA-IR, which was consistent with the conclusions of previous studies [43,44]. The mechanism of exercise that improved HOMA-IR mainly reflected in these aspects: (1) Exercise could relieve IR via the depletion of the metastasis-associated lung adenocarcinoma transcript 1 (MALAT1). MALAT1 was demonstrated to promote insulin sensitivity and increase insulin secretion. Moreover, MALAT1 was demonstrated to possess the ability to promote hepatic steatosis and IR [45]. (2) During exercise, skeletal muscle can release many muscle cytokines (IL-6, IL-10, IL-15). These muscle cytokines play an important anti-inflammatory role by regulating the maturation and redistribution of natural killer cells, promoting the activation of immune T cells, and improving skeletal muscle inflammation and IR [46]. Exercise can promote the expression of anti-inflammatory cytokine and inhibit the expression of pro-inflammatory cytokines, inhibit the IKK/NF-KB signal transduction pathway, downregulate the signal transduction of the Toll-like receptors, reduce the phosphorylation of JNK and expression of TNF-α, so as to reduce fat content and improve skeletal muscle inflammation and insulin resistance [47].

The following limitations of the meta-analysis need to be taken into account: (1) There is a significant heterogeneity with respect to the included outcomes. Although various subgroups (e.g., intervention duration and exercise modalities) and sensitivity analyses were performed to explore heterogeneity, significant heterogeneity was still clear and may be caused by sample size, exercise intensity, etc. (2) The number of included studies in the subgroups was relatively small. Although the results of the subgroup analysis reflect the significance of different modalities in different outcomes, the overall results still need to be confirmed by including more well-designed studies. (3) The stability of some outcomes may be affected by publication bias. It is hoped that more well-designed studies could further expand the results of the meta-analysis in the future.

## 5. Conclusions

Exercise can effectively improve cardiometabolic risk factors in obese adolescents with sedentary behavior. For obese adolescents who want to lose weight and improve cardiorespiratory fitness, combined aerobic and resistance training and high-intensity interval training are the optimal choices. For obese adolescents with high blood lipids, aerobic training can be regarded as a primary exercise modality to reduce high risk of cardiovascular diseases. For obese adolescents with insulin resistance, combined aerobic and resistance training can be considered to reduce high risk of diabetes. It is hoped that more high-quality studies would further expand the meta-analysis results and demonstrate the optimal exercise frequency and intensity in the future treatment of cardiometabolic risk factors in obese adolescents with sedentary behavior.

## Figures and Tables

**Table 1:** Search strategy on Pubmed.

#1	Search “Pediatric Obesity”[Mesh]
#2	Search ((((((((((((Adolescent Overweight[Title/Abstract]) OR (Obesity in Adolescence[Title/Abstract])) OR (Adolescent Obesity)[Title/Abstract])) OR (Infantile Obesity[Title/Abstract])) OR (Childhood Obesity[Title/Abstract])) OR (Obesity, Pediatric[Title/Abstract])) OR (Childhood Onset Obesity[Title/Abstract])) OR (Obesity, Childhood[Title/Abstract])) OR (Infant Overweight[Title/Abstract])) OR (Overweight, Infant[Title/Abstract])) OR (Childhood Overweight[Title/Abstract])) OR (Obesity, Infantile[Title/Abstract])) OR (Obesity, Child[Title/Abstract])
#3	Search #1 OR #2
#4	Search (((Exercise [Title/Abstract]) OR (Physical Activity [Title/Abstract])) OR (Sport [Title/Abstract])) OR (Training [Title/Abstract])
#5	Search #3 AND #4

**Table 2:** Characteristics of the studies included in the meta-analysis.

Study	Country	Characteristics of Patients				Intervention			Outcomes	Quality Assessment
		Sample Size (EXP/CT)	Gender (M/F)	Age (Years) (Mean ± SD)	Total Time	Frequency	Duration	Exercise Program		
Dai, 2006 [12]	China	64 (33/31)	None	8–12	60 min	5/weeks	12 weeks	Aerobic combined resistance training	②③④⑤	3
Benson, 2008 [13]	New Zealand	78 (37/41) EX:dropped 5 CT:dropped 3	EX:37 (22/15) CT:41 (24/17)	EX:12.3 ± 1.3 CT:12.2 ± 1.3	/	2/weeks	8 weeks	Resistance training	①②③⑤⑥	5
Karacabey, 2009 [14]	Turkey	40 (20/20)	EX:20(20/0) CT:20 (20/0)	EX:11.8 ± 0.5 CT:11.2 ± 0.8	30–60 min	3/weeks	12 weeks	Aerobic training (60–65% HRR)	①②③	3
TJØNNA, 2009 [15]	Norway	54 (28/26) EX:dropped 8 CT:dropped 4	EX:28(14/14) CT:26 (12/14)	EX:13.9 ± 0.3 CT:14.2 ± 0.3	40 min	2/weeks	12 weeks	High-intensity interval training (90–95% of HRmax)	①②④⑥	3
Sun, 2011 [16]	China	51 (29/22)	EX:29 (12/17) CT:22 (9/13)	13.6 ± 0.7	60 min	4/weeks	10 weeks	Aerobic training (40–60% VO_2_max)	①②③④⑤⑥⑦	3
Cao, 2012 [17]	China	40 (20/20)	EX:20(20/0) CT:20 (20/0)	13–15	50–60 min	2/weeks	8 weeks	High-intensity interval training (90–95% of HRmax)	①⑥	3
Seo, 2012 [18]	Korea	20 (10/10)	EX:10(10/0) CT:10 (10/0)	EX:14.7 ± 0.5 CT:14.6 ± 1	60 min	3/weeks	8 weeks	Yoga training (40–60% HRR)	①②③④⑤⑦	3
Racil.A, 2013 [19]	Tunisia	23 (11/12)	EX:11(0/11) CT:12 (0/12)	EX:15.6 ± 0.7 CT:15.9 ± 1.2	/	3/weeks	12 weeks	High-intensity interval training (100–110% of MAS)	②③④⑤⑥⑦	3
Racil.B, 2013 [19]	Tunisia	23 (11/12)	EX:11(0/11) CT:12 (0/12)	EX:16.3 ± 0.52 CT:15.9 ± 1.2	/	3/weeks	12 weeks	Aerobic interval training (70–80% of MAS)	②③④⑤⑥⑦	3
Chen.A, 2015 [20]	China	30 (15/15)	EX:15(15/0) CT:15(15/0)	EX:14.1 ± 3.1 CT:14.4 ± 3.2	60 min	3/weeks	8 weeks	Aerobic training (60% VO_2_max)	①⑥	3
Chen.B, 2015 [20]	China	30 (15/15)	EX:15(15/0) CT:15(15/0)	EX:13.9 ± 2.2 CT:14.4 ± 3.2	60 min	3/weeks	8 weeks	Resistance training	①⑥	3
Chen.C, 2015 [20]	China	30 (15/15)	EX:15(15/0) CT:15(15/0)	EX:14.2 ± 3.8 CT:14.4 ± 3.2	60 min	3/weeks	8 weeks	Aerobic combined resistance training	①⑥	3
Monteiro, 2015 [21]	Brazil	34 (18/16)	EX:18(10/8) CT:16(8/8)	EX:11 ± 1.02 CT:11.04 ± 1.9	50 min	3/weeks	20 weeks	Aerobic training (65–85% VO_2_max)	②③④⑤	4
Vasconcellos, 2016 [22]	Brazil	20 (10/10)	EX:10(8/2) CT:10(6/4)	EX:14.1 ± 1.3 CT:14.8 ± 1.4	60 min	3/weeks	12 weeks	Soccer training	①②③④⑤⑥⑦	6
Son, 2017 [7]	Korea	40 (20/20)	EX:20(0/20) CT:20(0/20)	EX:15 ± 1 CT:15 ± 1	60 min	3/weeks	12 weeks	Aerobic combined resistance training (40–50% HRR- 50–60% HRR- 60–70% HRR)	①⑦	3
Zhang, 2017 [23]	China	40 (20/20)	/	EX:13.78 ± 0.67 CT:14.71 ± 0.79	35 min	7/weeks	/	Acute exercise	①②③④⑤	3
Bharath, 2018 [6]	Korea	40 (20/20)	EX:20(0/20) CT:20(0/20)	EX:14.6 ± 1 CT:14.8 ± 1	60 min	5/weeks	12 weeks	Aerobic combined resistance training (40–50% HRR- 50–60% HRR- 60–70% HRR)	①⑥⑦	4
Chuensiri, 2018 [24]	Thailand	32 (16/16) EX:dropped 5 CT:dropped 5	EX:11(11/0) CT:11(11/0)	EX:11 ± 0.3 CT:10.6 ± 0.3	23 min	3/weeks	12 weeks	High-intensity interval training (90% peak power output)	①②③④⑤⑥	3
Kim, 2020 [5]	Korea	48 (24/24)	EX:24(0/24) CT:24(0/24)	EX:15 ± 1 CT:15 ± 1	50 min	5/weeks	12 weeks	Rope jumping	①⑦	3

Abbreviations: EX: Experimental group (Exercise group); CT: Control group; M/F: male/female; HRmax: Maximal heart rate; HRR: Heart rate reserve; VO^2^max: Maximal oxygen uptake; MAS: Maximal aerobic speed. Outcomes: ① BMI; ② HDL-C; ③ LDL-C; ④ TG; ⑤ TC; ⑥ VO_2_peak; ⑦ HOMA-IR.

**Table 3:** Subgroup analyses in obese adolescents.

Outcomes	Subgroup	Potential Factors	Included Studies	Sample Size	95% Confidence Intervals	Heterogeneity	*p*-Value
BMI	Intervention duration	Duration < 12weeks	7	268	−1.01[−1.77,−0.26]	I^2^ = 45%; *p* = 0.09	*p* = 0.009
		Duration ≥ 12weeks	8	292	−1.07[−1.26,−0.87]	I^2^ = 94%; *p* < 0.00001	*p* < 0.00001
	Exercise modality	AT	5	189	−3.7[−5.76,−1.64]	I^2^ = 83%; *p* < 0.0001	*p* = 0.0004
		RT	2	97	−0.92[−0.54,2.39]	I^2^ = 0%; *p* = 0.51	*p* = 0.22
		CART	3	110	−1.21[−1.62,−0.8]	I^2^ = 83%; *p* = 0.003	*p* < 0.00001
		HIIT	3	104	−0.63[−0.87,−0.4]	I^2^ = 0%; *p* = 0.61	*p* < 0.00001
HDL	Intervention duration	Duration < 12weeks	3	138	0.07[−0.27,0.41]	I^2^ = 38%; *p* = 0.2	*p* = 0.68
		Duration ≥12weeks	8	268	0.87[0.03,1.72]	I^2^ = 89%; *p* < 0.00001	*p* = 0.06
	Exercise modality	AT	4	147	0.74[0.32,1.8]	I^2^ = 88%; *p* < 0.00001	*p* = 0.17
		RT	1	67	0.32[−0.17,0.8]	/	*p* = 0.2
		CART	1	64	0.54[0.04,1.03]	/	*p* = 0.06
		HIIT	4	108	0.85[−0.89,2.59]	I^2^ = 93%; *p* < 0.00001	*p* = 0.34
LDL	Intervention duration	Duration < 12weeks	3	138	−0.05[−0.38,0.29]	I^2^ = 26%; *p* = 0.26	*p* = 0.79
		Duration ≥12weeks	7	226	−1.09[−1.70,−0.48]	I^2^ = 76%; *p* = 0.0003	*p* = 0.0005
	Exercise modality	AT	4	145	−0.89[−1.73,−0.06]	I^2^ = 81%; *p* = 0.001	*p* = 0.04
		RT	1	67	−0.13[−0.61,0.35]	/	*p* = 0.59
		CART	1	64	−0.43[−0.92,0.07]	/	*p* = 0.09
		HIIT	3	68	−1.35[−2.61,−0.09]	I^2^ = 80%; *p* = 0.006	*p* = 0.04
TG	Intervention duration	Duration < 12weeks	2	71	−0.56[−1.03,−0.08]	I^2^ = 0%; *p* = 0.50	*p* = 0.02
		Duration ≥12weeks	7	228	−0.43[−1.15,0.29]	I^2^ = 84%; *p* < 0.00001	*p* = 0.25
	Exercise modality	AT	3	105	−0.71[−1.11,−0.31]	I^2^ = 0%; *p* = 0.96	*p* = 0.0005
		CART	1	64	−0.24[−0.73,0.26]	/	*p* = 0.35
		HIIT	4	110	−0.38[−1.86,1.1]	I^2^ = 91%; *p* < 0.00001	*p* = 0.62
TC	Intervention duration	Duration < 12weeks	3	138	−0.06[−0.28,0.39]	I^2^ = 0%; *p* = 0.49	*p* = 0.73
		Duration ≥12weeks	6	186	−0.86[−1.56,−0.16]	I^2^ = 78%; *p* = 0.0003	*p* = 0.02
	Exercise modality	AT	3	105	−0.46[−0.85,−0.07]	I^2^ = 29%; *p* = 0.24	*p* = 0.02
		RT	1	67	0.11[−0.37,0.59]	/	*p* = 0.66
		CART	1	64	−0.13[−0.62,0.36]	/	*p* = 0.6
		HIIT	3	68	−1.31[−2.96,0.35]	I^2^ = 88%; *p* = 0.0002	*p* = 0.12
VO_2_peak	Intervention duration	Duration < 12weeks	6	244	3.16[1.18,5.14]	I^2^ = 69%; *p* = 0.007	*p* = 0.002
		Duration ≥12weeks	6	170	3.49[0.86,6.11]	I^2^ = 97%; *p* < 0.00001	*p* = 0.009
	Exercise modality	AT	3	101	3.61[1.72,5.51]	I^2^ = 53%; *p* = 0.12	*p* = 0.0002
		RT	2	93	0.72[−1.50,2.93]	I^2^ = 31%; *p* = 0.23	*p* = 0.53
		CART	2	52	8.48[7.47,9.49]	I^2^ = 89%; *p* = 0.003	*p* < 0.00001
		HIIT	5	168	2.5[2.10,2.89]	I^2^ = 89%; *p* < 0.00001	*p* < 0.00001
HOMA−IR	Intervention duration	Duration < 12weeks	2	71	−0.13[−0.87,0.62]	I^2^ = 52%; *p* = 0.15	*p* = 0.74
		Duration ≥12weeks	6	194	−2.77[−4.41,−1.14]	I^2^ = 94%; *p* < 0.00001	*p* = 0.0009
	Exercise modality	AT	3	119	−0.72[−1.72,0.27]	I^2^ = 84%; *p* = 0.002	*p* = 0.15
		CART	2	80	−6.52[−7.68,−5.37]	I^2^ = 0%; *p* = 0.54	*p* < 0.00001
		HIIT	2	46	−0.9[−1.89,0.08]	I^2^ = 60%; *p* = 0.11	*p* = 0.07

Abbreviations: BMI: Body mass index; HDL−C: High-density liptein cholesterol; LDL-C: Low-density liptein cholesterol; TG: Triglyceride; TC: Total cholesterol; VO_2_peak: Peak oxygen uptake; HOMA-IR: Homeostatic Model Assessment-Insulin Resistance; AT: Aerobic training; RT: Resistance training; CART: Combined aerobic and resistance training; HIIT: High-intensity interval training.

**Table 4:** Egger’s test of the included studies.

Outcomes	*n*	Std.Err	t	*p* > |t|	95% CI	Interval
BMI	15	1.03	−2.13	0.05	−4.43	0.03
HDL-C	12	3.90	0.18	0.86	−7.99	9.37
LDL-C	11	2.48	−2.29	0.05	−11.28	−0.08
TG	10	3.08	−1.70	0.13	−12.33	1.88
TC	10	1.99	−3.50	0.01	−11.58	−2.38
VO_2_peak	12	1.98	0.08	0.94	−4.27	4.57

## Data Availability

The data used in this study are available with this article.
